# Residual Neuromuscular Deficits Following Anterior Cruciate Ligament Reconstruction Revealed by Phase-Specific Force, Electromyographic Complexity and Inter-Limb Asymmetries in Flywheel Squat

**DOI:** 10.3390/s26185745

**Published:** 2026-09-10

**Authors:** Sara Ledesma-Clopés, Víctor Illera-Domínguez, Víctor Toro-Román, Carla Pérez-Chirinos Buxadé, Sara González-Millán, Lluís Albesa-Albiol, Vinyet Solé-González, Bruno Fernández-Valdés

**Affiliations:** 1 Research Group in Technology Applied to High Performance and Health, Department of Health Sciences, TecnoCampus, Universitat Pompeu Fabra, 08302 Mataró, Spain; sledesma@tecnocampus.cat (S.L.-C.); vtoro@tecnocampus.cat (V.T.-R.); cperezchirinosb@tecnocampus.cat (C.P.-C.B.); sgonzalezm@tecnocampus.cat (S.G.-M.); lalbesa@tecnocampus.cat (L.A.-A.); vsole@edu.tecnocampus.cat (V.S.-G.); bfernandez-valdes@tecnocampus.cat (B.F.-V.); 2 National Institute of Physical Education of Catalonia (INEFC), 08038 Barcelona, Spain; 3 Faculty of Communications and Social Sciences, University of San Jorge, Villanueva de Gállego, 50830 Zaragoza, Spain

**Keywords:** eccentric, concentric, entropy, muscle activation, injury, knee

## Abstract

**Background:** Anterior cruciate ligament (ACL) injuries induce neurophysiological alterations that may persist after rehabilitation, affecting motor control and neuromuscular coordination. This study aimed to examine residual neuromuscular deficits in team-sport athletes who had undergone ACL reconstruction by assessing phase-specific force production, electromyographic (EMG) signal complexity, and inter-limb asymmetries. **Methods:** Thirteen competitive team-sport athletes (men: n = 5, 24.2 ± 2.9 years; women: n = 8, 21.5 ± 1.9 years) participated in this cross-sectional study. Force production and surface EMG activity of the vastus lateralis (VL) and biceps femoris (BF) were recorded during an iso-inertial squat. Force data were analyzed globally and according to the concentric (CON) and eccentric (ECC) phases. Inter-limb asymmetries were calculated, and linear analyses were applied to force and EMG variables, while EMG complexity was assessed using sample entropy (SampEn). **Results:** Significant inter-limb differences were observed during the ECC phase, with lower peak and mean force values in the ACL-reconstructed limb (ACLR; *p* < 0.05), whereas no statistically significant inter-limb differences were identified in the global or CON analyses. Conventional EMG measures showed no statistically significant inter-limb differences. VL SampEn values were descriptively lower in the ACLR than in the contralateral non-reconstructed limb (ACLN), but this difference did not reach statistical significance (*p* = 0.063; Hedges’ *g* = −0.67). Inter-limb asymmetries showed substantial inter-individual variability, particularly across movement phases. Athletes following ACL reconstruction exhibited residual deficits in eccentric force production, whereas conventional EMG activation did not differ significantly between limbs. Phase-specific force analysis may therefore identify residual ECC deficits that are not apparent in global or concentric analyses. Although nonlinear EMG metrics may provide complementary information on neuromuscular organization beyond that captured by conventional amplitude-based measures, the present SampEn findings should be considered exploratory and hypothesis-generating rather than evidence of a confirmed alteration in neuromuscular complexity. Larger, adequately powered studies incorporating robustness and sensitivity analyses are required to determine the relevance of entropy-based EMG measures following ACL reconstruction.

## 1. Introduction

Anterior cruciate ligament (ACL) rupture is one of the most prevalent and clinically significant knee injuries among physically active young individuals, with reported incidence rates ranging from 0.03 to 0.42 ACL ruptures for every 1000 athlete-exposures, depending on sex, competitive level, and sporting discipline [1]. Its etiology is multifactorial, involving both modifiable and non-modifiable risk factors [1]. Among the modifiable factors, impaired neuromuscular control, defined as altered coordination between the central nervous system and the musculature responsible for knee stabilization [2], has been identified as a key contributor to increased mechanical loading and a greater susceptibility to ACL injury.

Following ACL rupture, neurophysiological changes such as disrupted proprioceptive feedback, delayed reflex responses, and altered motor control strategies have been widely described [2,3]. These adaptations may involve cortical reorganization and an increased reliance on non-ligamentous sensory afferents [4]. Even following ACL reconstruction, inter-limb asymmetries in muscle activation, reaction times, motor strategies, and postural control often persist [2] and have been associated with an increased risk of reinjury [5]. Therefore, restoration of ligament integrity does not necessarily imply restoration of neuromuscular function.

Neuromuscular variability (NMV) has emerged as a key concept for understanding motor system reorganization following ACL injury. Biological systems inherently exhibit variability [6], and human movement can be conceptualized as a complex, dynamic, and nonlinear system [7]. This variability is reflected in fluctuations in motor behavior, muscle activation patterns [8], spatiotemporal parameters during repetitive task execution [9], and intra-repetition irregularity [10,11]. From a dynamical systems perspective, functional variability represents the organism’s adaptive capacity to explore, adapt, and stabilize efficient motor solutions [12]. Reduced variability may indicate reduced motor flexibility, whereas excessive variability may reflect system instability [12]. Following lower-limb injury, the motor system tends to constrain its degrees of freedom, thereby reducing its capacity to adapt to external perturbations [13].

In sports, where performance relies on continuous adaptation to dynamic environments, NMV has emerged as a relevant marker of neuromuscular control and functional adaptability [14]. Variability in muscle activation patterns provides valuable information on coordinative flexibility, load-sharing mechanisms, and the capacity to respond effectively to task-specific demands [14,15]. In this context, assessing NMV may provide important insights into persistent neuromuscular alterations and compensatory motor strategies following ACL injury [15,16]. Recent evidence in ACL-reconstructed athletes further suggests that nonlinear measures of movement variability may reveal alterations in motor control not captured by conventional linear performance metrics [17].

Surface electromyography (EMG) enables the characterization of muscle activation patterns and the electrophysiological processes underlying movement and force production [18,19]. In individuals with ACL injury, alterations in mean EMG frequency, amplitude, and activation timing have been reported [20], suggesting changes in motor unit recruitment and neuromuscular control strategies. However, conventional amplitude-based EMG analyses have inherent limitations, as they may fail to capture the temporal organization and adaptive dynamics of NMV.

Nonlinear analytical approaches allow the quantification of dynamic properties that cannot be detected using traditional linear metrics [21,22]. Entropy-based analyses have been widely used to estimate the irregularity and complexity of physiological time series [7,23]. In particular, Sample Entropy (SampEn) provides a robust measure of temporal complexity, showing reduced dependence on time-series length and greater stability against noise [24,25]. In the neuromuscular context, changes in SampEn may reflect alterations in NMV, as it captures modifications in the temporal organization of muscle recruitment and motor system coordination. Therefore, SampEn represents a promising tool for investigating persistent neuromuscular adaptations following ACL reconstruction.

In recent years, iso-inertial training has received increasing attention due to its capacity to induce eccentric overload and optimize neuromuscular adaptations [26,27]. In individuals following ACL reconstruction, the inclusion of iso-inertial exercises within rehabilitation programs has been associated with improvements in muscular endurance, eccentric power, and reductions in inter-limb asymmetries [27]. However, the assessment of force production following ACL reconstruction continues to rely primarily on linear parameters, such as mean force, peak force, and inter-limb asymmetry indices, with inter-limb asymmetry thresholds of <5–10% commonly used as benchmarks for return to sport [28]. Nevertheless, these linear indicators remain insufficient for characterizing the dynamic organization of the neuromuscular system and the underlying NMV [29,30].

To date, no studies have simultaneously examined phase-specific force production, electromyographic temporal complexity, and inter-limb asymmetries to investigate residual neuromuscular adaptations following ACL reconstruction during iso-inertial flywheel tasks. Therefore, the aim of this study was to investigate residual neuromuscular deficits in competitive team-sport athletes following ACL reconstruction through an integrated assessment of phase-specific force production, electromyographic temporal complexity, and inter-limb asymmetries during the flywheel squat task. It was hypothesized that the ACL-reconstructed limb (ACLR) would exhibit lower EMG temporal complexity and force production than the contralateral non-reconstructed limb (ACLN), resulting in greater inter-limb asymmetries and reflecting persistent neuromuscular reorganization despite rehabilitation and return to sport.

## 2. Materials and Methods

### 2.1. Participants

The sample comprised 8 women (age 21.5 ± 1.9 years; height 164.2 ± 3.4 cm; body mass 63.8 ± 8.6 kg; time since surgery 3.4 ± 2.3 years) and 5 men (age 24.2 ± 2.9 years; height 184.4 ± 9.2 cm; body mass 79.9 ± 10.1 kg; time since surgery 2.7 ± 1.4 years). Regarding injury laterality, ACL reconstruction involved the right knee in 8 participants (61.5%) and the left knee in 5 participants (38.5%). Seven participants (53.8%) sustained the ACL injury in their dominant limb, whereas 6 (46.2%) sustained the injury in their non-dominant limb.

ACL reconstruction was performed using a hamstring tendon autograft in 7 participants and a bone–patellar tendon–bone autograft in 6 participants. All participants completed a physiotherapist-supervised rehabilitation program and returned to sport after a mean of 12.5 ± 4.1 months. At the time of assessment, all participants were competing at the regional level.

Sample size was estimated a priori using G*Power software (version 3.1.9.7; Heinrich-Heine-Universität Düsseldorf, Düsseldorf, Germany) for a repeated-measures ANOVA with two within-subject conditions (ACLR and ACLN), assuming a large effect size (f = 0.50), an α level of 0.05, and a statistical power of 0.80. The a priori analysis indicated a required sample size of 28 participants; however, recruitment constraints limited the final sample to 13 athletes. A post hoc analysis based on the observed effect size (f = 0.53) yielded a statistical power of 0.54. Given that the final sample did not reach the a priori estimated sample size and that the achieved statistical power was below the conventional threshold of 0.80, the findings of the present study should be regarded as preliminary and interpreted with appropriate caution. Further adequately powered studies with larger samples are warranted to confirm these findings.

The inclusion criteria were: (i) being older than 18 years, (ii) having undergone unilateral ACL reconstruction, (iii) being actively competing at the time of assessment, and (iv) having returned to full competition at least one month before data collection. Exclusion criteria included: (i) having undergone surgical intervention for more than one knee injury, (ii) a history of hip or ankle surgery, and (iii) any pathology or medical condition contraindicating physical activity.

In addition, participants were instructed to refrain from performing intense physical exercise during the 48 h prior to the assessment, as well as from consuming caffeine, energy drinks, or any other stimulant substances during the 24 h preceding the testing session.

Written informed consent was obtained from all participants prior to participation. The study protocol was approved by the TecnoCampus (Universitat Pompeu Fabra) Ethics Committee (approval number: 6/2024) and conducted in accordance with the Declaration of Helsinki.

### 2.2. Study Design

The study employed a cross-sectional within-subject design. The experimental protocol involved the assessment of force production and EMG activity during the flywheel squat task to compare neuromuscular responses between limbs.

All participants attended a single experimental session in a controlled laboratory environment. Before data collection, they performed a standardized warm-up consisting of 5 min of submaximal exercise on a cycle ergometer, followed by dynamic joint mobility exercises targeting the lower limbs. Subsequently, participants completed a familiarization session with the iso-inertial system and the flywheel squat exercise to ensure proper technique and minimize learning effects during testing. This familiarization phase formed part of the warm-up protocol.

**Figure 1:**
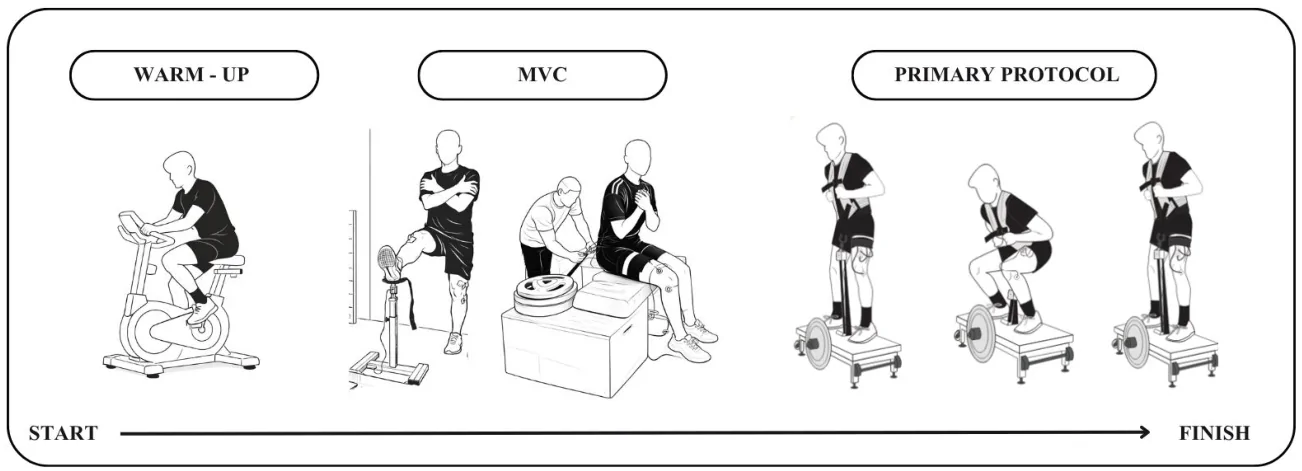
Schematic representation of the experimental protocol, including a standardized warm-up, a maximum voluntary contraction (MVC) assessment, and the primary flywheel squat exercise performed using an inertial training device.

Following the warm-up and familiarization, maximal voluntary contraction (MVC) assessments were performed, which were subsequently used for EMG signal normalization. Thereafter, participants completed the main protocol consisting of the execution of the flywheel squat using an iso-inertial system (Figure 1). Throughout the protocol, force production and EMG activity were recorded simultaneously using independent acquisition systems. Mechanical and EMG data were subsequently analyzed separately, as no hardware-based temporal synchronization between the two acquisition systems was performed.

The experiments were conducted under controlled environmental conditions, with temperatures ranging between 20 and 25 °C and relative humidity between 65 and 75%, while maintaining the execution of each test within a similar time window throughout the day.

### 2.3. Experimental Protocol

#### 2.3.1. Flywheel Squat Test

The flywheel squat was selected as the experimental task because of its high eccentric loading demands and its ability to reproduce neuromuscular loading patterns relevant to rehabilitation and return to sport following ACL reconstruction. The exercise was performed using the Acceleration Squat Excellence system (NeuroExcellence, Braga, Portugal), which incorporates two unilateral force platforms and a rotational encoder to record force signals and angular displacement, respectively. Force signals were sampled at 180 Hz, corresponding to the native acquisition frequency of the force platforms integrated into the system. This sampling rate was considered adequate for characterizing force production during a controlled, multi-joint movement such as the flywheel squat, as the relevant frequency content of the ground reaction force signal was expected to remain well below the corresponding Nyquist limit of 90 Hz [31].

Participants stood on the platforms with their feet shoulder-width apart and were connected to the system through a harness adjusted at hip level. From an upright standing position, they performed an eccentric phase by descending to approximately 90° of knee flexion, followed by a concentric phase executed at maximal velocity to accelerate the flywheel.

The protocol consisted of 30 consecutive repetitions performed with moment of inertia of 0.05 kg·m^2^. Participants were instructed to perform all repetitions with maximal voluntary effort during both the concentric and eccentric phases of the movement. Standardized verbal encouragement was continuously provided throughout the protocol to promote consistent maximal effort. Given the repeated maximal-effort nature of the task, fluctuations in mechanical output across repetitions were considered part of the individual performance response during the exercise. Accordingly, within-set consistency of force production was additionally characterized using the coefficient of variation (CV) calculated across the repetitions retained for analysis.

The inertial load of 0.05 kg·m^2^ was selected as a moderate inertia to allow high concentric execution velocities while eliciting substantial eccentric demands, consistent with the load–velocity characteristics of lower-body resistance exercise and the neuromuscular demands of flywheel exercise [32,33].

During the exercise, force production and EMG activity were recorded simultaneously. The rotational encoder signal was used to identify the movement reversal points and segment each repetition into eccentric and concentric phases. This segmentation enabled the independent analysis of phase-specific mechanical variables associated with the exercise.

#### 2.3.2. EMG Assessment

EMG activity was recorded using the portable mDurance Solutions SL (mDurance Solutions S.L., Granada, Spain) system, with a sampling frequency of 1024 Hz. Surface electrodes were placed bilaterally over the vastus lateralis (VL) and biceps femoris (BF) muscles according to SENIAM recommendations [34]. Prior to electrode placement, the skin was cleaned to improve electrode–skin contact and minimize potential recording artifacts. Electrodes were aligned with the direction of the underlying muscle fibers, and standardized placement procedures were followed to promote consistent positioning over the target muscle and minimize potential signal contamination from adjacent muscles.

For EMG signal normalization, maximal voluntary contractions (MVC) were performed (Figure 1). Maximum quadriceps activation was assessed through an isometric knee extension at 90° of knee flexion, a position known to facilitate high torque production and neuromuscular activation of the knee extensor apparatus [35]. Hamstring MVC was assessed through an isometric knee flexion at 20° of knee flexion with the hip positioned at 90°, as this configuration increases the musculotendinous length of the hamstrings and allows a sensitive assessment of their maximal force-generating capacity [33]. Each test consisted of three 5-s repetitions, with 60 s of recovery between attempts. The highest EMG amplitude recorded for each muscle was used as the reference for signal normalization. The selection of these positions aimed to maximize voluntary activation and minimize variability associated with the angular dependency of force production.

During the execution of the flywheel squat test, EMG activity from the VL and BF muscles was simultaneously recorded to characterize muscle activation patterns and NMV during movement. The acquired signals were subsequently used for the linear and nonlinear analyses described in the data processing section.

### 2.4. Data Analysis

#### 2.4.1. Signal Processing

EMG signals were processed using MATLAB R2024b (The MathWorks, Inc., Natick, MA, USA). The recorded signals were filtered using a fourth-order Butterworth band-pass filter (10–500 Hz) to attenuate low-frequency motion-related components and high-frequency noise. Zero-phase filtering was applied using the MATLAB filtfilt function to preserve the temporal characteristics of the EMG signal. Subsequently, the signals were full-wave rectified and normalized to the maximum value obtained during the corresponding MVC [36].

To delimit the EMG interval analyzed, the electromyographic recording was synchronized using the mDurance application (version 2.5.2; mDurance, Granada, Spain) with video acquired at 60 frames·s^−1^. Frame-by-frame inspection was used to identify the temporal boundaries of the 28 included repetitions, from the onset of the downward movement of the first repetition to the recovery of the upright position after the final repetition. The video was used solely to establish these boundaries and not to segment the EMG signal by repetition or movement phase.

The rotational encoder signal was used exclusively to identify the onset and termination of each repetition and the reversal points delimiting the concentric (CON) and eccentric (ECC) phases of the mechanical data. No external synchronization pulses, shared triggers, or hardware-based synchronization procedures were used to temporally align the mechanical recordings obtained from the force platform and rotational encoder with the synchronized EMG–video recording. Consequently, no direct temporal correspondence was established between instantaneous EMG activity and repetition- or phase-specific mechanical data.

Of the 30 maximal-effort repetitions, the first and final repetitions were excluded *a priori* from both the mechanical and EMG analyses. Force data from the remaining 28 repetitions were visually inspected for signal integrity and retained for both limbs of all 13 participants.

#### 2.4.2. Asymmetry Analysis

Inter-limb asymmetry was calculated for force- and EMG-derived variables to quantify differences between the ACLR and ACLN. The asymmetry index was calculated using the following equation [37]:

Asymmetry (%) = [(ACLN − ACLR)/ACLN] × 100

where ACLR represents the anterior cruciate ligament-reconstructed limb and ACLN represents the non-reconstructed contralateral limb. The sign of the asymmetry index was retained to preserve the direction of the inter-limb difference. Accordingly, positive values indicated higher values in the ACLN relative to the ACLR, whereas negative values indicated higher values in the ACLR relative to the ACLN. Thus, the magnitude of the index quantified the relative inter-limb difference, while its sign identified the direction of the asymmetry.

For force-derived outcomes, mean force values were first obtained for each participant and limb according to movement phase (global, CON, and ECC). The asymmetry index was subsequently calculated from these limb-specific mean values. Thus, force asymmetry was derived from participant-level aggregated values for each movement phase rather than calculated separately for each individual repetition.

For EMG-derived outcomes, asymmetry indices were calculated separately for the VL and BF using the corresponding participant-level values for each EMG-derived variable. For SampEn, the asymmetry index was calculated from the single representative value obtained for each participant, muscle, and limb from the continuous EMG interval encompassing the 28 repetitions retained for analysis.

As an additional quality-control step, observations showing particularly large inter-limb asymmetry values (>50%) were individually reviewed. The underlying limb-specific values were inspected and, for EMG-derived variables, the corresponding normalized signals were visually re-examined to identify evident signal-quality, normalization, or processing anomalies. No evident anomalies attributable to these procedures were identified in the observations retained for analysis. This inspection was considered a quality-control procedure rather than a formal assessment of measurement reliability.

#### 2.4.3. EMG Signal Quality Assurance

To reduce movement-related artifacts during the dynamic flywheel squat task, the EMG cables were securely fixed to the participant to limit cable displacement and minimize movement-induced disturbances at the electrode–skin interface [38]. Cable routing was arranged to avoid excessive tension or oscillation that could compromise electrode stability during movement.

EMG signal quality was visually monitored throughout data acquisition. Recordings were inspected for transient disturbances indicative of signal contamination, including abrupt signal discontinuities, excessive baseline fluctuations, movement-related artifacts, or other identifiable sources of interference [38]. When a clear transient artifact occurred during an individual repetition, the affected repetition was excluded from subsequent analysis rather than corrected or interpolated. This procedure was adopted to prevent artifact-contaminated data from influencing either conventional EMG-derived variables or entropy estimates. Only repetitions showing stable recordings without identifiable signal contamination throughout the analyzed interval were retained for subsequent analysis.

Electrode–skin impedance was not quantitatively measured during data collection. Nevertheless, standardized skin-preparation and electrode-placement procedures based on SENIAM recommendations were followed to optimize electrode–skin contact and reduce potential signal contamination. Although electrode alignment and placement were selected to minimize crosstalk from adjacent muscles, no formal crosstalk assessment was performed. Therefore, the possibility of residual signal contamination cannot be completely excluded and is acknowledged as a methodological limitation.

Although the protocol was not specifically designed to induce neuromuscular fatigue, fatigue-related changes in EMG signal characteristics across consecutive repetitions were not formally quantified. Therefore, a potential temporal effect associated with repeated maximal-effort contractions cannot be completely excluded and should be considered when interpreting the entropy estimates.

#### 2.4.4. Nonlinear Analysis

To assess the temporal complexity of the EMG signals, Sample Entropy (SampEn) was calculated from the rectified EMG signals using custom scripts in R, following the method described by Richman and Moorman [39]. An embedding dimension of *m* = 2 and a tolerance threshold of *r* = 0.2 × SD of the signal were used, consistent with established methodological recommendations for physiological time-series analysis [39] and previous applications of entropy-based methods to surface EMG signals [40].

Participants initially performed three repetitions to accelerate the flywheel, followed by 30 maximal-effort repetitions. The acceleration repetitions were not included in the analysis. Of the 30 maximal-effort repetitions, the first and final repetitions were excluded *a priori*. SampEn was therefore calculated from the continuous EMG interval encompassing the remaining 28 repetitions, yielding a single representative value for each participant, muscle, and limb.

The selected EMG interval was analyzed as a single uninterrupted time series. Individual repetitions were not analyzed separately or concatenated, and no phase-specific SampEn values were calculated. The EMG recordings were processed independently of the rotational encoder, which was used exclusively to identify repetitions and concentric and eccentric phases within the mechanical data. Signal quality was assessed as described in the EMG Signal Quality Assurance Section.

SampEn estimates may be influenced by signal length, preprocessing procedures, residual noise, and the selected *m* and *r* parameters. Although the parameter settings used in the present study were based on established methodological recommendations, their robustness was not evaluated using alternative signal lengths, processing choices, parameter configurations, or repetition-by-repetition analyses. Accordingly, the entropy findings should be regarded as exploratory and interpreted with appropriate caution.

### 2.5. Statistical Analysis

Statistical analyses were performed to compare both limbs (ACLR and ACLN). Data normality was assessed using the Shapiro–Wilk test and visual inspection of Q–Q plots.

Force-related variables, including mean force, peak force, and the coefficient of variation (CV), were not normally distributed (*p* < 0.05) and were analyzed using the Wilcoxon signed-rank test. The CV was calculated to quantify the intra-individual variability of force production across repetitions. For linear and nonlinear EMG-derived variables, normality of the within-participant differences between ACLR and ACLN was assessed. As no substantial deviations from normality were identified, paired-samples *t*-tests were used for inter-limb comparisons.

To assess the temporal evolution of mechanical performance, mean concentric and eccentric force values were normalized to the mean of the first three analyzed repetitions for each participant and limb. Separate linear mixed-effects models were fitted for the concentric and eccentric phases. Repetition was entered as a continuous fixed effect, together with limb and the repetition-by-limb interaction, while participant was included as a random intercept. Model estimates are reported with their corresponding 95% confidence intervals and *p*-values.

Descriptive statistics are reported as median [interquartile range] or mean ± standard deviation, as appropriate. Effect sizes were calculated as *r* for non-parametric comparisons (*r* = *Z*/√*N*, where *N* represents the total number of observations) and Hedges’ *g* for parametric analyses. Effect sizes are reported together with their 95% confidence intervals (95% CI).

All statistical analyses were performed using R (version 4.5.3; R Core Team, Vienna, Austria) within the RStudio environment (version 2026.01.1; RStudio Team, Boston, MA, USA). Statistical significance was set at *p* < 0.05.

## 3. Results

### 3.1. Force

Normalized mean force decreased significantly across repetitions during both the concentric and eccentric phases (effect of repetition: both *p* < 0.001; Supplementary Figure S1). No repetition-by-limb interaction was observed in either the concentric (*p* = 0.747) or eccentric phase (*p* = 0.643), indicating no evidence of differential temporal patterns of force output between the ACLR and ACLN.

In the overall analysis across movement phases (global, CON, and ECC), statistically significant inter-limb differences were only observed during the eccentric phase, whereas no significant differences were found in the global or concentric phases (Table 1).

**Figure 2:**
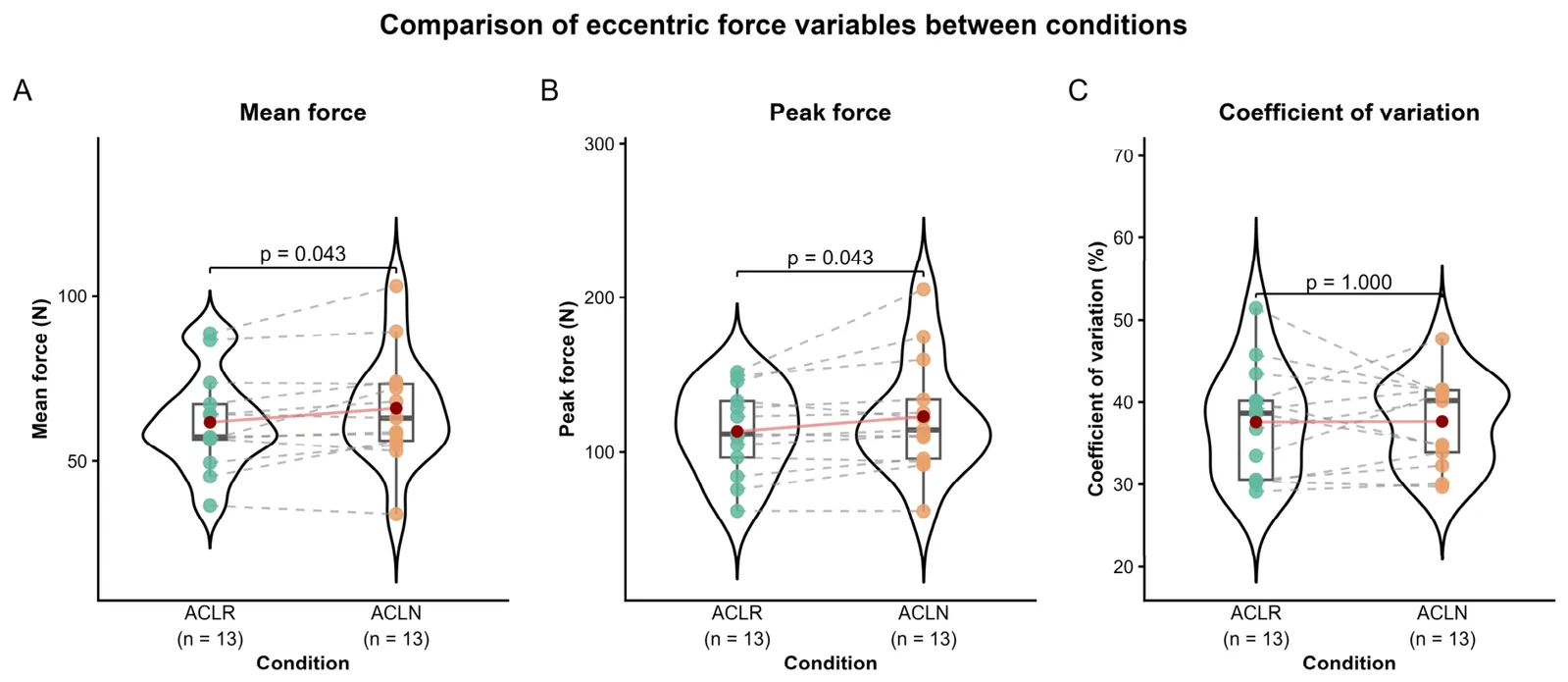
Inter-limb comparison of eccentric force variables during the flywheel squat task. Mean force (**A**), peak force (**B**), and coefficient of variation (**C**) during the eccentric phase were compared between the ACL-reconstructed limb (ACLR) and the contralateral non-reconstructed limb (ACLN). Each point represents an individual participant (n = 13), and dashed lines connect paired observations from the same participant.

During the ECC phase, statistically significant inter-limb differences were observed in peak force (Figure 2). Specifically, peak force was significantly lower in the ACLR compared with the ACLN (ACLR = 111.89 [96.48, 132.98] N; ACLN = 114.56 [95.74, 133.96] N), with a Hodges–Lehmann difference of −6.57 N (*p* = 0.043). A strong positive association between limbs was observed according to Kendall’s τ coefficient (τ = 0.846).

Similarly, mean force was lower in the ACLR compared with the ACLN in most cases (ACLR = 57.36 [56.56, 67.23] N; ACLN = 63.08 [56.18, 73.30] N), representing a signed difference of −6.57 N (*p* = 0.043). A strong positive association between limb-specific values was also observed (Kendall’s τ = 0.718).

In contrast, regarding the CV, no statistically significant differences were observed between limbs (ACLR = 38.68 [30.57, 40.17]%; ACLN = 40.17 [33.90, 41.44]%; absolute difference = −0.14%; *p* = 1.000), and a moderate positive association between limb-specific CV values was observed (Kendall’s τ = 0.487).

Paired Wilcoxon signed-rank tests revealed significant inter-limb differences in both mean and peak force (both *p* = 0.043), with an effect size of r = −0.65 (95% CI [−0.88, −0.15]) for both variables. In contrast, no significant inter-limb difference was observed in the CV (*p* = 1.000; r = −0.01; 95% CI [−0.56, 0.54]).

In the global and CON phases, no statistically significant differences were identified between limbs (Table 1). Although peak and mean force values were descriptively lower in the ACLR than in the ACLN, these differences did not reach statistical significance (*p* > 0.05) in either phase. Likewise, no statistically significant inter-limb differences were observed in the CV across the analyzed phases.

**Figure 3:**
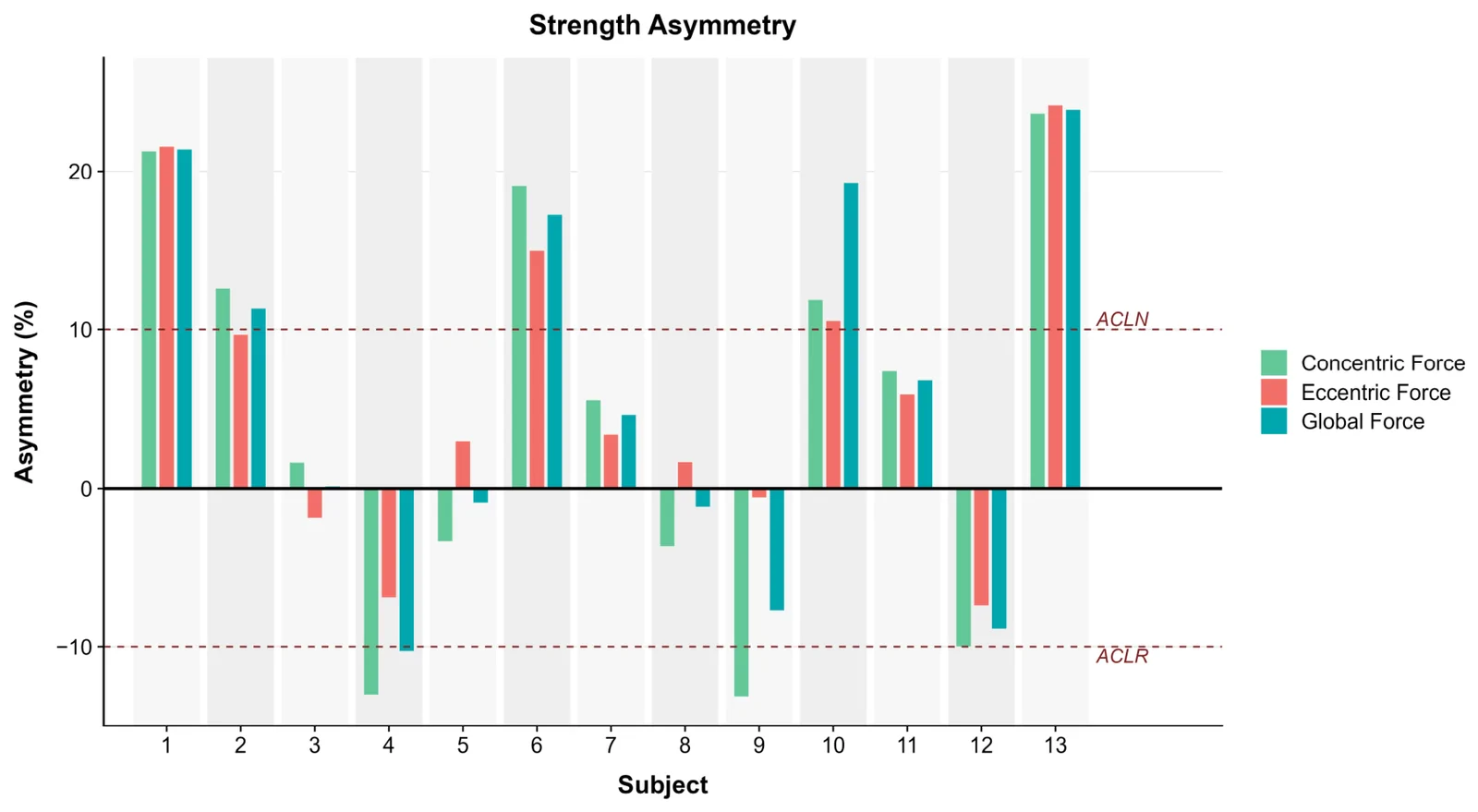
Participant-specific directional inter-limb force asymmetry across the concentric and eccentric phases and the overall movement. Green bars represent concentric-phase force, coral bars represent eccentric-phase force, and turquoise bars represent global force calculated over the entire movement. Each cluster of three bars represents one participant. Positive values indicate greater force production by the non-reconstructed limb (ACLN), whereas negative values indicate greater force production by the ACL-reconstructed limb (ACLR). The solid horizontal line represents perfect inter-limb symmetry (0%), while the dashed horizontal lines indicate the predefined ±10% asymmetry thresholds.

Figure 3 shows a clear inter-subject heterogeneity in asymmetry values between ACLR and ACLN, both in magnitude and direction, across the three evaluated phases (global, CON, and ECC). A high proportion of participants exhibited positive asymmetry values greater than 10%, indicating a predominance of the ACLN in terms of force production. However, negative asymmetry values with magnitudes greater than 10% were also observed in some participants, reflecting better performance of the ACLR.

Some participants displayed particularly large inter-limb asymmetry values (>50%). Quality-control inspection of these extreme observations did not identify evident signal-quality, normalization, or processing anomalies that could account for their magnitude.

### 3.2. Surface Electromyography

**Figure 4:**
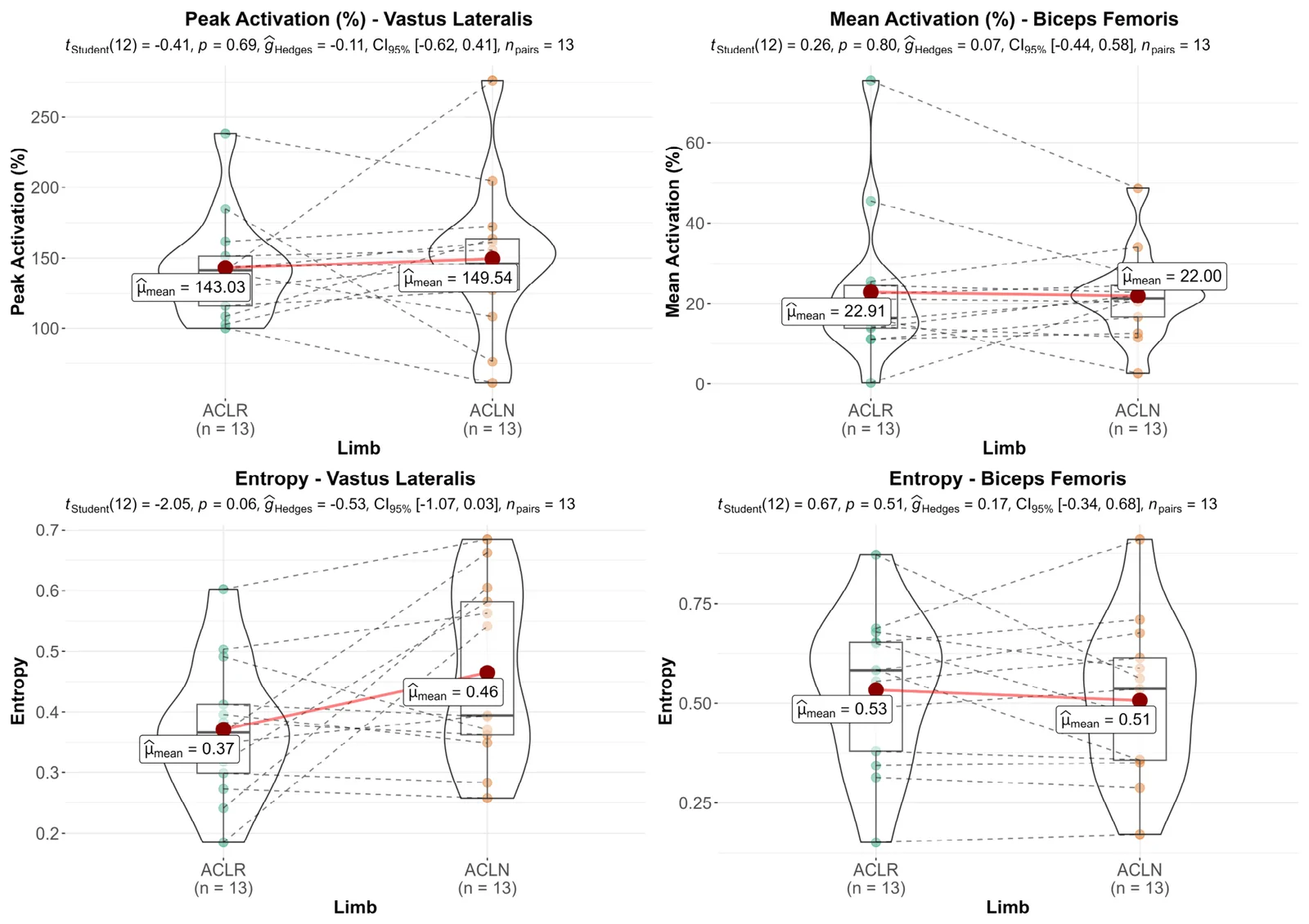
Inter-limb comparison of conventional and nonlinear EMG-derived variables between the ACL-reconstructed limb (ACLR) and the contralateral non-reconstructed limb (ACLN). Peak vastus lateralis (VL) activation, mean biceps femoris (BF) activation, and Sample Entropy (SampEn) for both muscles are presented. Each point represents one participant (n = 13), and dashed lines connect paired observations from the same participant. EMG-derived variables were calculated over the continuous global movement interval encompassing the 28 repetitions retained for analysis; no phase-specific EMG variables were derived.

Figure 4 presents normalized EMG amplitude and EMG signal complexity. All EMG amplitude and entropy variables reported in this section were calculated over the continuous global movement interval encompassing the 28 repetitions retained for analysis; no phase-specific EMG variables were derived. No statistically significant differences were observed between the ACLR and ACLN for any of the analyzed outcomes.

In the VL, peak activation during the squat did not differ significantly between conditions (ACLR: 143.03 ± 37.47%; ACLN: 149.54 ± 54.25%), with a mean difference of −6.51% (*p* = 0.687; Hedges’ *g* = −0.13). Similarly, mean BF activation did not differ significantly between the ACLR (22.91 ± 19.00%) and the ACLN (22.00 ± 11.15%), with a difference of −0.91% (*p* = 0.799; Hedges’ *g* = 0.07).

Regarding signal entropy, no statistically significant inter-limb differences were observed in the BF (ACLR: 0.53 ± 0.19; ACLN: 0.51 ± 0.20; *p* = 0.513; Hedges’ *g* = 0.13). In the VL, SampEn was descriptively lower in the ACLR (0.37 ± 0.11) than in the contralateral limb (0.46 ± 0.15), with a mean inter-limb difference of −0.09. However, this difference was not statistically significant (*p* = 0.063; Hedges’ *g* = −0.67).

**Figure 5:**
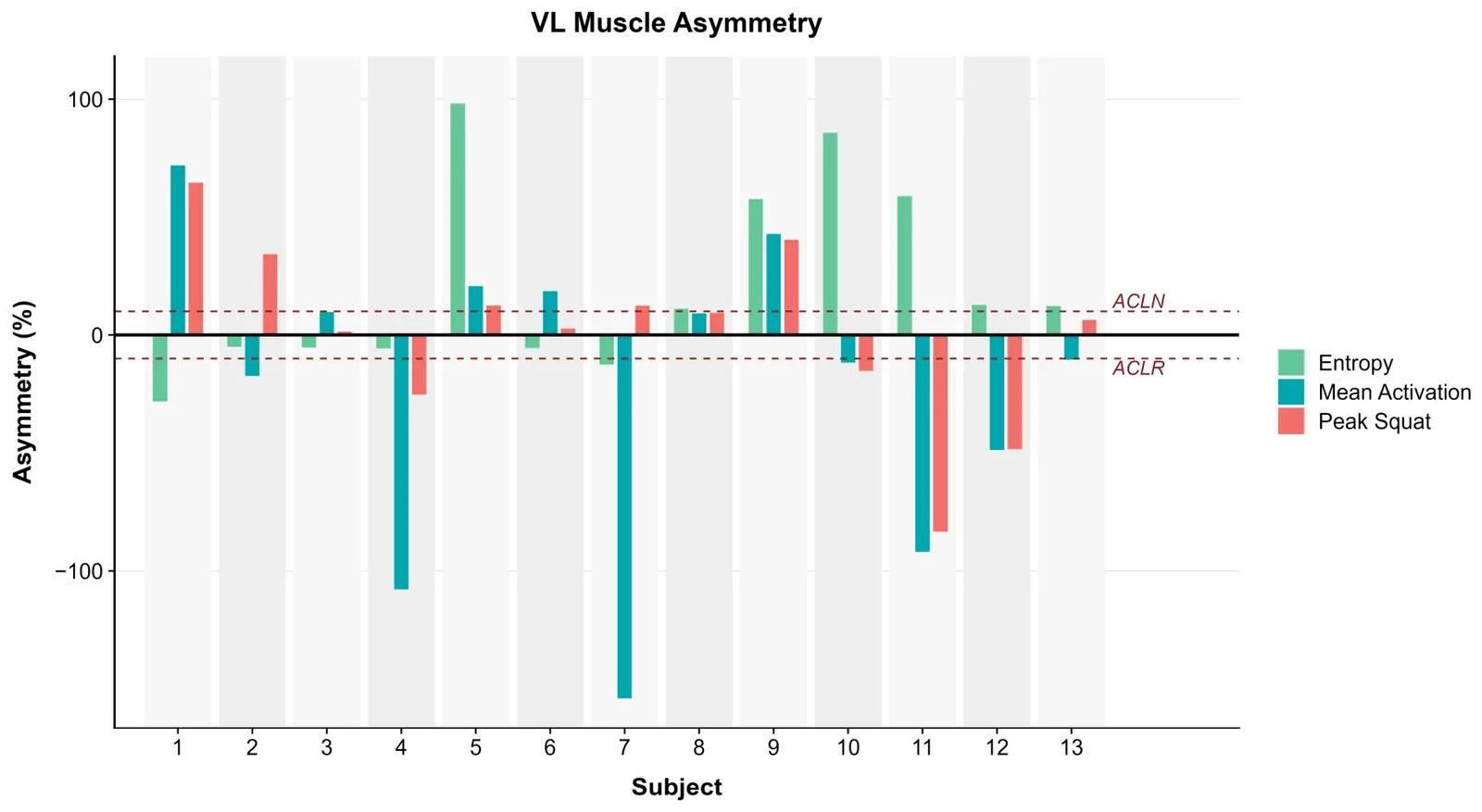
Participant-specific directional inter-limb asymmetry in vastus lateralis (VL) electromyographic activity. Green bars represent mean EMG activation, coral bars represent peak EMG activation during the flywheel squat, and turquoise bars represent Sample Entropy (SampEn). Each cluster of three bars corresponds to one participant. Positive values indicate greater values in the non-reconstructed limb (ACLN), whereas negative values indicate greater values in the ACL-reconstructed limb (ACLR). The solid horizontal line denotes perfect inter-limb symmetry (0%), and the dashed horizontal lines denote the predefined ±10% asymmetry thresholds.

**Figure 6:**
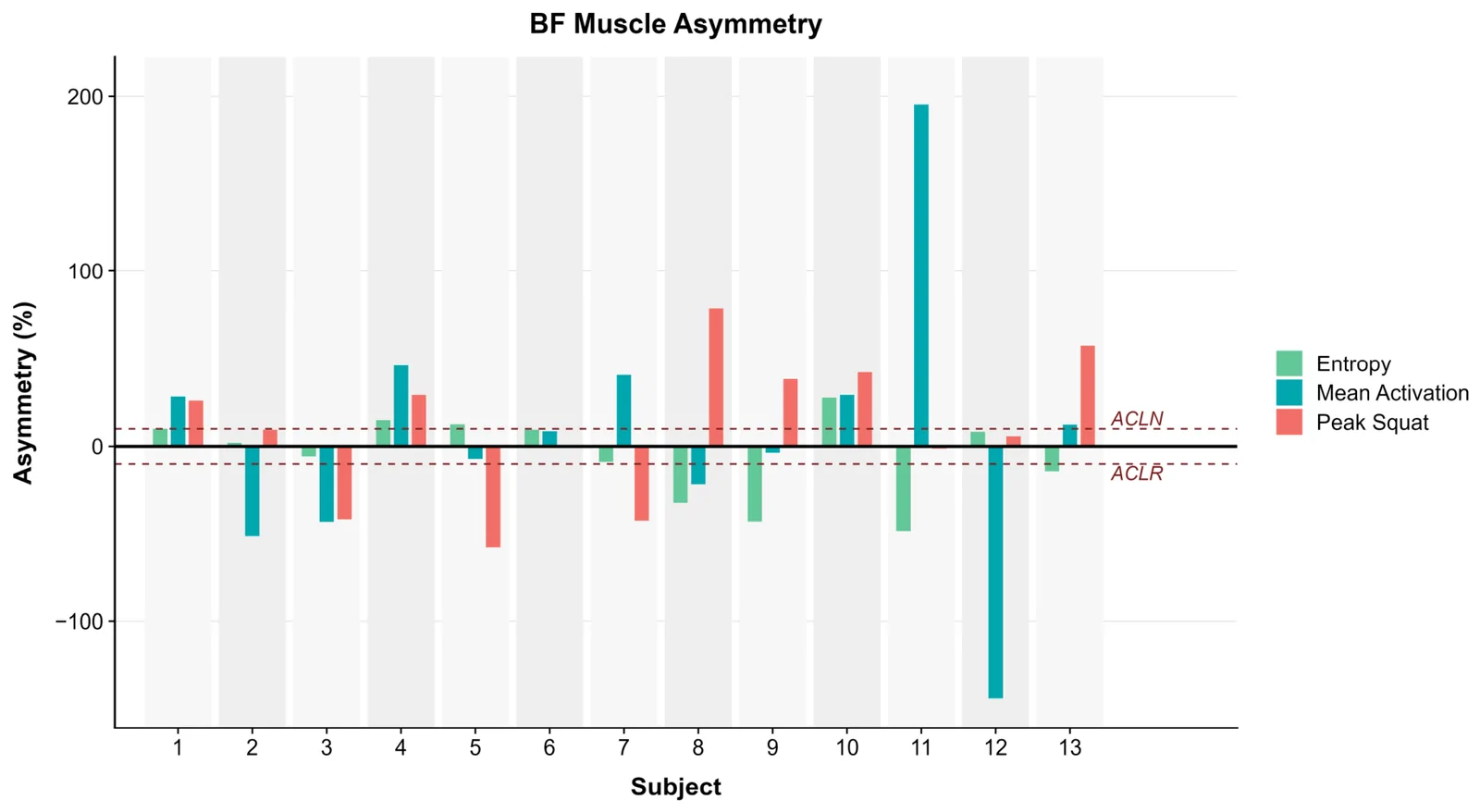
Participant-specific directional inter-limb asymmetry in biceps femoris (BF) electromyographic activity. Green bars represent mean EMG activation, coral bars represent peak EMG activation during the flywheel squat, and turquoise bars represent Sample Entropy (SampEn). Each cluster of three bars corresponds to one participant. Positive values indicate greater values in the contralateral non-reconstructed limb (ACLN), whereas negative values indicate greater values in the ACL-reconstructed limb (ACLR). The solid horizontal line denotes perfect inter-limb symmetry (0%), the dashed horizontal lines denote the predefined ±10% asymmetry thresholds.

When descriptively analyzing subject-specific asymmetries in the VL and BF muscle groups (Figure 5 and Figure 6), the distribution pattern was found to be heterogeneous, with most participants showing at least one variable with asymmetry values exceeding the ±10% threshold. In some cases, asymmetries were positive (greater activation in the ACLN), whereas in others they were negative (greater activation in the ACLR), highlighting the heterogeneity of post-injury activation patterns.

In the VL muscle (Figure 5), most participants exhibited asymmetry values greater than ±10%, with some variables exceeding ±50%, particularly those related to entropy and peak activation. The asymmetry ranges included both positive and negative values, reflecting a wide inter-individual variability in functional differences between the ACLR and ACLN. However, the highest asymmetry values were predominantly observed in the positive direction, indicating greater entropy in the ACLN in several participants.

In the BF muscle (Figure 6), inter-limb asymmetries showed a lower magnitude compared with those observed in the VL muscle. Although elevated values were identified in specific participants, most measurements were distributed within a narrower range of variation. The deviations included both positive and negative values, without showing a predominant direction toward greater activation in either the ACLR or ACLN. This distribution suggests lower asymmetry in muscle activity between the two limbs. Likewise, the three analyzed variables (peak activation, mean activation, and entropy) showed lower inter-individual dispersion in the BF compared with the VL.

## 4. Discussion

The main findings of this study indicate that, despite returning to sport, athletes following ACL reconstruction exhibited significant inter-limb differences in eccentric force production during the iso-inertial squat, whereas conventional EMG-derived variables showed no statistically significant inter-limb differences. Nonlinear EMG analysis revealed lower VL entropy values in ACLR, although this difference did not reach statistical significance. Accordingly, the entropy results should be interpreted as exploratory and hypothesis-generating rather than as evidence of a confirmed neuromuscular deficit. Collectively, these observations highlight the potential value of integrating phase-specific mechanical assessment with conventional and nonlinear EMG metrics to characterize neuromuscular function following ACL reconstruction.

Although the protocol was not specifically designed to induce neuromuscular fatigue, repetition-by-repetition analysis revealed a significant decline in force output during both the CON and ECC phases. However, the absence of a repetition-by-limb interaction indicates no evidence of a differential temporal pattern between ACLR and ACLN. Thus, the within-set reduction appears to reflect a general response to the repeated maximal-intended-effort task rather than a limb-specific phenomenon. This pattern does not appear to fully explain the inter-limb differences observed in ECC force production. Nevertheless, because mechanical output was not maintained throughout the set, potential fatigue- or pacing-related effects should be considered when interpreting force and neuromuscular variability outcomes [13,41,42].

The reduced force output observed during the ECC phase is consistent with previous evidence reporting persistent deficits in knee extensor function following ACL injury, particularly during tasks requiring high levels of deceleration control and load absorption [15]. This finding is particularly relevant given the critical role of the ECC phase in maintaining dynamic knee stability and managing the mechanical loads associated with high-demand sporting actions [43]. The presence of these deficits in rehabilitated athletes suggests that neuromuscular function may not be fully restored, even after meeting conventional return-to-sport criteria.

Stańczak et al. [2] demonstrated that ACL injury not only compromises ligament structural integrity but also induces persistent alterations in sensorimotor control, including afferent integration, reflexive mechanisms, and motor planning. The loss of afferent input from ligamentous mechanoreceptors may consequently impair the nervous system’s capacity to regulate muscle activation and coordinate motor responses under complex functional demands. These alterations may be particularly relevant during the ECC phase, where precise deceleration control and load absorption require substantial neuromuscular coordination. In this context, the force deficits observed in ACLR may reflect persistent neurophysiological adaptations following ACL injury and reconstruction.

Descriptive analysis revealed substantial inter-individual variability in the magnitude and direction of force asymmetries between ACLR and ACLN across the evaluated phases (global, CON, and ECC). This observation is consistent with Guan et al. [28], who showed that inter-limb asymmetries may persist even after return to sport. Although thresholds of 10–15% have traditionally been proposed to define clinically relevant asymmetries, current evidence increasingly questions the validity of universal cut-off values, as highlighted by Bourne et al. [44] and Dos’ Santos et al. [45]. Such heterogeneity may reflect individualized compensatory strategies, complicating the establishment of standardized criteria for clinical interpretation. These observations therefore reinforce the need for individualized assessment approaches that account for each athlete’s functional profile.

The magnitude of some individual asymmetries also warrants careful consideration, as values exceeding 50% were observed in some participants. Although quality-control inspection did not identify evident signal-quality, normalization, or processing anomalies that could explain these extreme values, this does not establish their physiological origin. Large asymmetries may reflect genuine inter-limb differences but may also be influenced by intra-individual variability, residual measurement noise, and the mathematical properties of the asymmetry calculation. The present design does not allow these physiological and methodological contributions to be fully disentangled. Therefore, extreme asymmetry values should not be interpreted as direct estimates of functional impairment but rather as indicators of substantial inter-individual heterogeneity.

A relevant aspect of this study is the phase-specific analysis of movement asymmetries. Examining asymmetries across the global, CON, and ECC phases provides a more nuanced characterization of each athlete’s functional profile. As highlighted by Bishop et al. [28], asymmetries may vary considerably depending on the task and movement phase; therefore, phase-specific monitoring may enhance the sensitivity of functional assessment. This approach may be especially informative in athletes following ACL reconstruction, as deficits may emerge selectively according to the mechanical and neuromuscular demands of each phase.

Regarding muscle activation, no statistically significant inter-limb differences were observed in conventional EMG-derived activation parameters. Specifically, VL peak activation and BF mean activation did not differ significantly between ACLR and ACLN, providing no evidence of inter-limb differences in activation magnitude following rehabilitation.

The observed pattern may be interpreted within the optimal variability framework proposed by Stergiou et al. [12], which suggests that healthy biological systems operate within an intermediate range of variability that facilitates adaptation to environmental demands. From this perspective, reduced variability has been associated with a diminished capacity to dynamically reorganize motor output in response to perturbations or changing task demands. Accordingly, the descriptively lower entropy values observed in the ACLR may be consistent with a more constrained temporal organization of neuromuscular output. However, given that the inter-limb difference in SampEn did not reach statistical significance and considering the limited statistical precision of the present study, this interpretation should be regarded as exploratory and hypothesis-generating and requires confirmation in larger, adequately powered studies.

Previous studies provide relevant context for interpreting these results. Hollman et al. [46] reported reduced nonlinear EMG-derived complexity of the vastus medialis in individuals with ACL injury, which was interpreted as reflecting alterations in neuromuscular control. Similarly, Davi et al. [47] observed reduced quadriceps neuromuscular complexity following ACL reconstruction and reported associations between lower entropy values and deficits in voluntary activation. Together, these studies support the potential value of complexity-based metrics for characterizing aspects of neuromuscular function not captured by conventional EMG measures. However, given the limited sample size and statistical power of this study, the current results are insufficient to determine whether comparable alterations in EMG complexity persist after return to sport.

The absence of significant inter-limb differences in conventional EMG-derived activation variables, together with the descriptive entropy pattern, suggests that activation magnitude and temporal organization may provide complementary information on neuromuscular function. Entropy-based metrics may therefore capture features of neuromuscular organization not reflected by conventional EMG measures, although their clinical relevance remains to be established.

Notably, participants were, on average, more than three years post-surgery and had fully returned to sport. The descriptively lower entropy values observed in this context provide a rationale for investigating whether differences in neuromuscular organization persist after return to sport in larger, adequately powered cohorts. However, the cross-sectional design and limited statistical precision preclude conclusions regarding the persistence or temporal evolution of these adaptations. Previous evidence of persistent alterations in sensorimotor integration and motor control following ACL reconstruction [46,47] provides a rationale for further investigation.

From an applied perspective, nonlinear metrics may complement conventional EMG assessment by characterizing the temporal organization of muscle activity rather than solely its magnitude. However, whether this complementary information improves the sensitivity or clinical utility of neuromuscular assessment following ACL reconstruction remains to be determined.

In contrast, the BF showed no significant inter-limb differences in either activation or entropy. The distinct descriptive patterns observed in the BF and VL may suggest muscle-specific responses following ACL reconstruction. However, the current evidence is insufficient to establish differential neuromuscular adaptations between muscle groups, warranting further investigation in larger studies.

Individualized analysis of muscle activation asymmetries revealed substantial inter-individual variability, particularly in the VL, where asymmetries greater than ±10% were observed in most participants and exceeded ±50% in some cases. The bidirectional distribution of these asymmetries further emphasizes the importance of athlete-specific interpretation rather than reliance solely on population-based symmetry criteria.

Taken together, these results support a more integrative approach to the assessment of athletes following ACL reconstruction. Conventional EMG measures, complexity-based metrics, and phase-specific force assessment characterize distinct aspects of neuromuscular performance and may therefore provide complementary information on individual functional profiles. Further research is needed to determine whether integrating these approaches provides additional clinical value for rehabilitation monitoring and return-to-sport decision-making.

Despite the relevance of these findings, several limitations should be acknowledged. The relatively small sample size reduced statistical precision and the ability to detect small-to-moderate inter-limb differences, particularly in nonlinear EMG-derived variables. Consequently, non-significant findings should not be interpreted as evidence of equivalence between limbs, and observed differences should be interpreted cautiously given the uncertainty surrounding their estimates. SampEn estimates may also be influenced by signal-processing procedures and parameter selection. Although the parameters used were selected according to established methodological recommendations and previous applications to surface EMG, no sensitivity analysis was performed to assess the robustness of SampEn estimates across alternative processing and parameter settings. The cross-sectional design precludes establishing causal relationships or determining the temporal evolution of the observed neuromuscular patterns throughout rehabilitation and after return to sport. Furthermore, although participants were instructed to perform each repetition with maximal voluntary effort, force output declined across the set, indicating that mechanical performance was not maintained throughout the protocol. This decline may limit the generalizability of the results to shorter maximal-effort tasks and should be considered when interpreting force production and neuromuscular variability. Finally, the assessment was restricted to a specific functional task and a limited number of muscles. Future adequately powered studies should incorporate broader neuromuscular assessments, different functional tasks, and longitudinal designs to determine the robustness and clinical relevance of these observations.

In addition, within-session reliability indices (e.g., intraclass correlation coefficients) were not computed for the asymmetry estimates, limiting the interpretation of particularly large individual values. Because SampEn was calculated from a continuous EMG interval encompassing 28 repetitions, temporal changes associated with the repeated-effort task may also have influenced entropy estimates.

Finally, only a single inertial load (0.05 kg·m^2^) was evaluated. Different loads may elicit distinct neuromuscular responses, limiting the generalizability of these results to other loading conditions.

## 5. Conclusions

The findings of this study indicate that neuromuscular recovery following ACL reconstruction may involve aspects that are not fully captured by conventional functional metrics. Phase-specific force analysis identified lower ECC force production in the reconstructed limb, whereas conventional EMG-derived activation variables showed no significant inter-limb differences. Nonlinear EMG analysis showed descriptively lower VL entropy values in the reconstructed limb; however, this difference did not reach statistical significance. Therefore, the entropy findings should be regarded as exploratory and hypothesis-generating rather than as evidence of a confirmed neuromuscular deficit.

Furthermore, the different patterns observed between muscle groups, together with the marked inter-individual variability in neuromuscular asymmetries, highlight the complexity and individual nature of recovery following ACL reconstruction. Collectively, these findings support the potential value of integrating phase-specific mechanical assessment with conventional and complexity-based EMG metrics to provide a more comprehensive characterization of neuromuscular function. However, the robustness and clinical relevance of complexity-based EMG measures remain to be established in larger, adequately powered studies.

## 6. Practical Applications

The incorporation of advanced neuromuscular analyses during rehabilitation following ACL reconstruction may provide relevant information for a more comprehensive evaluation of the athlete’s functional status. Phase-specific force analysis may help identify specific deficits that are not apparent in global assessments, potentially supporting more targeted interventions aligned with the functional demands of movement.

Similarly, complexity-based EMG metrics may complement conventional amplitude-based measures by providing additional information on the temporal organization of muscle activity. However, given the exploratory nature of the present entropy findings, the clinical utility of these metrics for identifying residual neuromuscular alterations following ACL reconstruction remains to be established.

Taken together, the integration of phase-specific force assessment with conventional and nonlinear EMG analyses may provide a broader framework for characterizing individual neuromuscular profiles during rehabilitation and return-to-sport assessment. Further research is required to determine whether this integrated approach provides additional value for clinical decision-making.

From an implementation standpoint, nonlinear EMG analyses can be computed from standard surface EMG recordings without additional equipment; however, their routine clinical adoption currently requires signal-processing expertise and standardized, validated analysis pipelines, which may limit their accessibility outside research settings in the short term.

## Figures and Tables

**Table 1:** Force production and variability metrics in the ACLR and ACLN during the flywheel squat task.

Variable	Phase	ACLR	ACLN	HL Difference	ES (r)	95% CI	*p*
Mean Force (N)	Global	62.95 [58.09, 66.08]	63.44 [61.71, 73.83]	−3.79	−0.388	[−1.03, 0.06]	0.162
	CON	62.69 [59.39, 68.74]	66.26 [58.71, 73.96]	−3.13	−0.330	[−0.89, 0.17]	0.235
Peak Force (N)	Global	114.06 [104.73, 133.74]	125.54 [109.91, 133.96]	−8.08	−0.485	[−1.04, 0.05]	0.081
	CON	114.06 [96.50, 133.74]	111.67 [108.37, 126.68]	−6.57	−0.397	[−0.94, 0.13]	0.152
Force CV (%)	Global	36.88 [31.11, 40.33]	36.65 [32.93, 39.22]	−0.54	−0.097	[−0.64, 0.39]	0.727
	CON	34.15 [28.95, 40.48]	35.02 [29.23, 37.66]	−0.34	−0.097	[−0.57, 0.45]	0.727
	ECC	38.68 [30.57, 40.17]	40.17 [33.90, 41.44]	−0.14	0.000	[−0.52, 0.5]	1.000

Values are presented as median [interquartile range]. Paired comparisons between the ACLR and ACLN were performed using the Wilcoxon signed-rank test. HL difference represents the Hodges–Lehmann estimator of the paired median difference between limbs (ACLR − ACLN). Effect size is reported as *r* and calculated as *r* = *Z*/√*N*. CI = confidence interval; CON = concentric phase; ECC = eccentric phase; CV = coefficient of variation.

## Data Availability

The raw data supporting the conclusions of this article will be made available by the authors on request.

## Supplementary Materials

The following supporting information can be downloaded at: https://www.mdpi.com/article/10.3390/s26185745/s1, Figure S1: Repetition-by-repetition force progression during the flywheel squat. Points and lines represent group mean concentric (A) and eccentric (B) force across 28 repetitions, normalized to the mean of repetitions 1–3; shaded areas denote 95% confidence intervals. ACLR, ACL-reconstructed limb; ACLN, contralateral non-reconstructed limb.

## Abbreviations

The following abbreviations are used in this manuscript:

ACL	Anterior cruciate ligament
ACLR	ACL-reconstructed limb
ACLN	Contralateral non-reconstructed limb
EMG	Electromyography
SampEn	Sample Entropy
CON	Concentric
ECC	Eccentric
NMV	Neuromuscular Variability
MVC	Maximum Voluntary Contraction
VL	Vastus Lateralis
BF	Biceps Femoris
CV	Coefficient of Variation
