# Supplementary material for: Residual Neuromuscular Deficits Following Anterior Cruciate Ligament Reconstruction Revealed by Phase-Specific Force, Electromyographic Complexity and Inter-Limb Asymmetries in Flywheel Squat
Source: Sensors (Basel). 2026 Sep 10;26(18):5745. doi: 10.3390/s26185745 (PMC13611060; doi:10.3390/s26185745)
Supplement: Supplementary file 1 [file sensors-26-05745-s001.zip › sensors-4470179-supplementary.pdf]

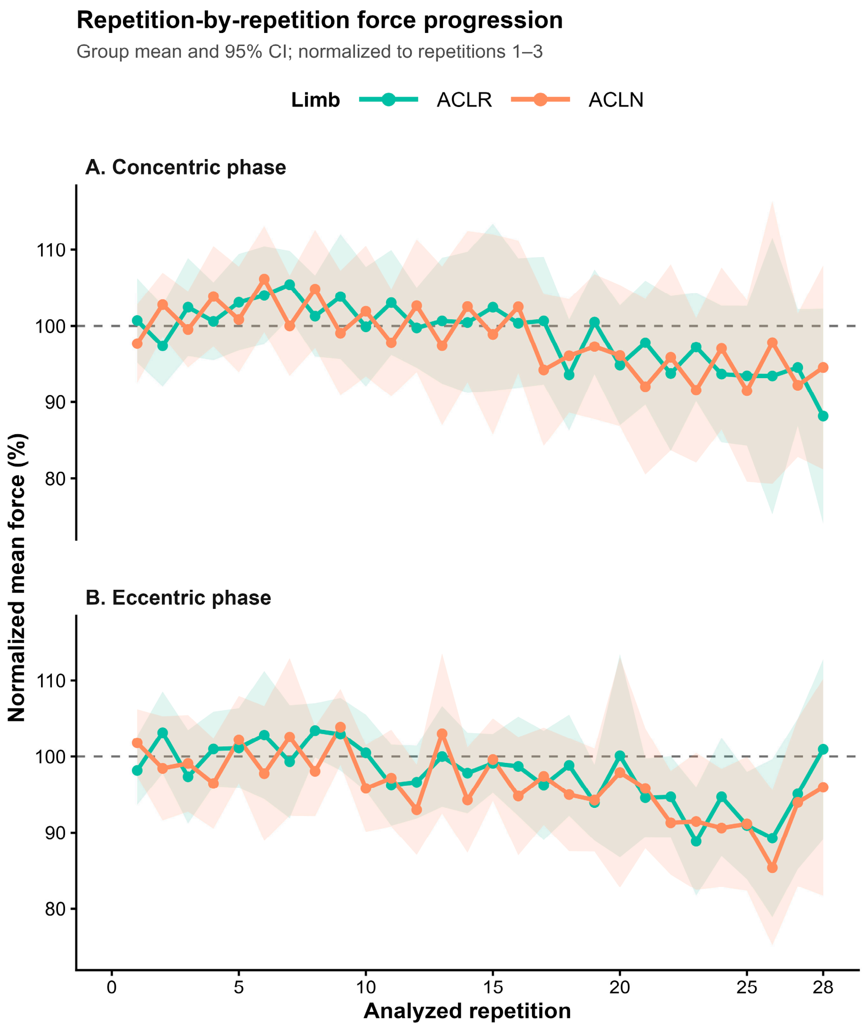

Supplementary Figure S1. Repetition-by-repetition force progression during the flywheel squat. Points and lines represent group mean concentric (A) and eccentric (B) force across 28 repetitions, normalized to the mean of repetitions 1–3; shaded areas denote 95% confidence intervals. ACLR, ACL-reconstructed limb; ACLN, contralateral non-reconstructed limb.
